# P-1475. Real-World Effectiveness and Safety of Cefiderocol in the Treatment of Patients with Serious Gram-negative Bacterial Infections: Results of the PROVE Chart Review Study

**DOI:** 10.1093/ofid/ofae631.1645

**Published:** 2025-01-29

**Authors:** Cornelius J Clancy, Oliver A Cornely, Christine M Slover, Sean T Nguyen, Frank H Kung, Stefano Verardi, Christopher M Longshaw, Stephen Marcella, Bin Cai

**Affiliations:** University of Pittsburgh, Pittsburgh, Pennsylvania; University of Cologne, Faculty of Medicine and University Hospital Cologne, Cologne, Nordrhein-Westfalen, Germany; Shionogi Inc., Florham Park, New Jersey; Shionogi Inc., Florham Park, New Jersey; Shionogi Inc, Florham Park, New Jersey; Shionogi, B.V., London, England, United Kingdom; Shionogi B.V., London, England, United Kingdom; Shionogi Inc, Florham Park, New Jersey; Shionogi Inc, Florham Park, New Jersey

## Abstract

**Background:**

The ongoing PROVE retrospective chart review study aims to assess real-world outcomes of cefiderocol treatment in patients with serious Gram-negative bacterial infections.

**Figure:**
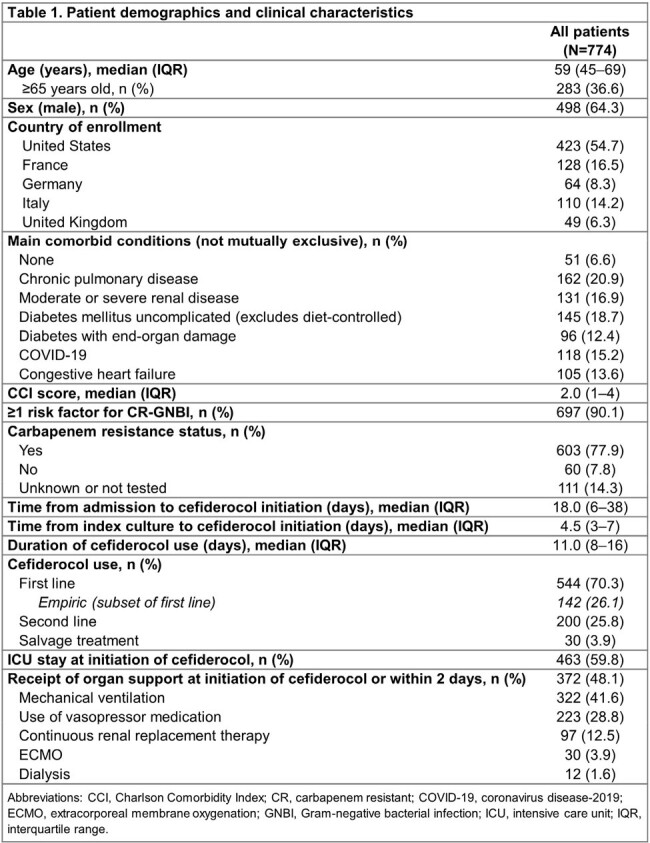

**Methods:**

This is an interim analysis of hospitalized patients with suspected or confirmed Gram-negative bacterial infections in Europe and the USA treated with cefiderocol for the first time for ≥72 hours (Nov 2020–Feb 2024). A total of 1000 patients are planned. Baseline demographics, clinical characteristics, cefiderocol use, clinical cure, clinical response at end of treatment (EOT), in-hospital all-cause mortality (ACM), and adverse drug reactions (ADRs) were assessed.

**Figure:**
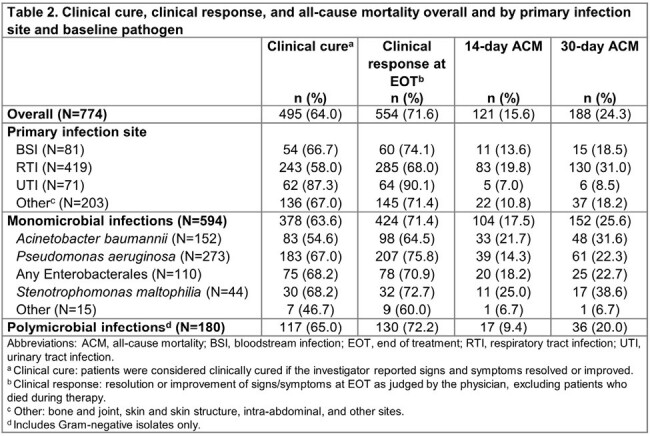

**Results:**

Of 774 patients analyzed, 498 (64.3%) were men and the median age (interquartile range [IQR]) was 59 (45–69) years (**Table 1**); 90.1% of patients had ≥1 risk factor for carbapenem-resistant infections. Overall, 59.8% were in the intensive care unit (ICU) and 48.1% received organ support. Cefiderocol was used as first-line therapy in 70.3% of patients; the median (IQR) duration was 11 (8–16) days. The most frequent infection site was the respiratory tract (RTI; 54.1%), and the most frequent species in monomicrobial infections was *Pseudomonas aeruginosa* (35.3%), followed by *Acinetobacter baumannii* (19.6%) (**Figure 1**). Overall, 64.0% of patients had clinical cure and 71.6% responded to cefiderocol treatment at EOT. ACM rates at days 14 and 30 were 15.6% and 24.3%, respectively (**Table 2**). Clinical cure and clinical response rates were 58.0% and 68.0% in patients with RTI, and 66.7% and 74.1% in patients with bloodstream infection (BSI), respectively. Among patients with RTI or BSI, 31.0% and 18.5% died by day 30, respectively. Among patients with *P. aeruginosa* infection, 67.0% and 75.8% had clinical cure and clinical response, and 22.3% died by day 30. In monomicrobial and polymicrobial infections, clinical cure rates were 63.6% and 65.0%, while ACM rates were 25.6% and 20.0% at day 30, respectively (**Table 2**). A total of 19 patients had 23 ADRs or serious ADRs, which led to discontinuation of cefiderocol for 11 events (**Table 3**).

**Figure:**
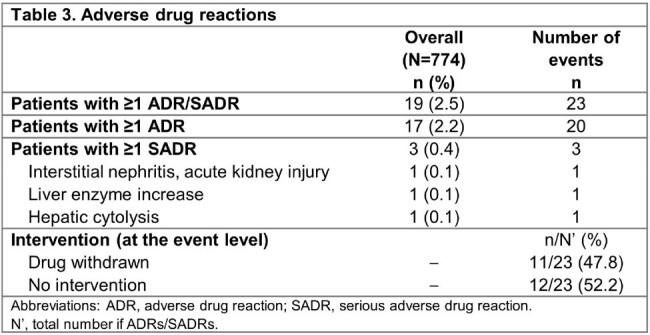

**Conclusion:**

Cefiderocol was efficacious and well tolerated for the treatment of serious Gram-negative bacterial infections in real-world settings in a patient population with high rates of ICU admission and organ support.

**Figure:**
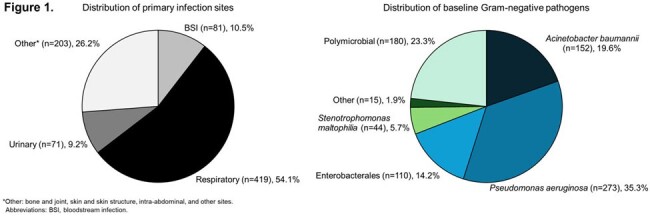

**Disclosures:**

**Cornelius J. Clancy, MD**, Cidara: Grant/Research Support|Gilead: Honoraria|Merck: Grant/Research Support|Scynexis: Advisor/Consultant|Shionogi: Advisor/Consultant|Venatorx: Advisor/Consultant **Oliver A. Cornely, Prof. Dr.**, Abbott: Honoraria|Abbvie: Advisor/Consultant|Abbvie: Honoraria|AiCuris: Advisor/Consultant|Akademie fur Infektionmedizin: Honoraria|Al-Jazeera Pharmaceuticals/Hikma: Honoraria|amedes: Honoraria|AstraZeneca: Honoraria|Basilea: Advisor/Consultant|Biocon: Advisor/Consultant|BMBF: Grant/Research Support|Boston Strategic Partners: Advisor/Consultant|CIdara: Advisor/Consultant|CIdara: Expert Testimony|CIdara: Grant/Research Support|CIdara: Participation on a DRC or DSMB|CoRe Consulting: Stocks/Bonds (Private Company)|Deutscher Arzteverlag: Honoraria|DZIF: Grant/Research Support|EasyRadiology: Stocks/Bonds (Private Company)|EU-DG RTD: Grant/Research Support|F2G: Grant/Research Support|Gilead: Advisor/Consultant|Gilead: Grant/Research Support|Gilead: Honoraria|Grupo Biotoscana/United Medical/Knight: Honoraria|GSK: Advisor/Consultant|GSK: Honoraria|IQVIA: Advisor/Consultant|IQVIA: Participation on a DRC or DSMB|Janssen: Advisor/Consultant|Janssen: Participation on a DRC or DSMB|Matinas: Advisor/Consultant|MedPace: Advisor/Consultant|MedPace: Grant/Research Support|MedPace: Participation on a DRC or DSMB|Medscape/WebMD: Honoraria|MedUpdate: Honoraria|Menarini: Advisor/Consultant|Moderna: Honoraria|Molecular Partners: Advisor/Consultant|MSD: Grant/Research Support|MSD: Honoraria|MSG-ERC: Advisor/Consultant|Mundipharma: Advisor/Consultant|Mundipharma: Grant/Research Support|Mundipharma: Honoraria|Noscendo: Honoraria|Noxxon: Advisor/Consultant|Octapharma: Advisor/Consultant|Octapharma: Grant/Research Support|Pardes: Advisor/Consultant|Partner Therapeutics: Advisor/Consultant|Patent: US18/562644|Paul-Martini-Stiftung: Honoraria|Pfizer: Advisor/Consultant|Pfizer: Grant/Research Support|Pfizer: Honoraria|PSI: Advisor/Consultant|PSI: Participation on a DRC or DSMB|Pulmocide: Participation on a DRC or DSMB|Sandoz: Honoraria|Scynexis: Advisor/Consultant|Scynexis: Grant/Research Support|Seqirus: Advisor/Consultant|Seqirus: Honoraria|Seres: Advisor/Consultant|Shionogi: Advisor/Consultant|Shionogi: Honoraria|streamedup!: Honoraria|The Prime Meridian Group: Advisor/Consultant|Touch Independent: Honoraria|Vitis: Honoraria **Christine M. Slover, PharmD**, Shionogi Inc.: Employee **Sean T. Nguyen, PharmD**, Shionogi Inc.: Employee **Frank H. Kung, PhD**, Shionogi Inc.: Employee **Stefano Verardi, MD**, Shionogi BV: Employee **Christopher M. Longshaw, PhD**, Shionogi BV: Employee **Stephen Marcella, MD, MPH**, Shionogi Inc.: Employee **Bin Cai, MD, PhD**, Shionogi Inc.: Employee

